# *Myosin phosphatase* Fine-tunes Zebrafish Motoneuron Position during Axonogenesis

**DOI:** 10.1371/journal.pgen.1006440

**Published:** 2016-11-17

**Authors:** Juliane Bremer, Michael Granato

**Affiliations:** Department of Cell and Developmental Biology, Perelman School of Medicine, University of Pennsylvania, Philadelphia, Pennsylvania, United States of America; University of Maine, UNITED STATES

## Abstract

During embryogenesis the spinal cord shifts position along the anterior-posterior axis relative to adjacent tissues. How motor neurons whose cell bodies are located in the spinal cord while their axons reside in adjacent tissues compensate for such tissue shift is not well understood. Using live cell imaging in zebrafish, we show that as motor axons exit from the spinal cord and extend through extracellular matrix produced by adjacent notochord cells, these cells shift several cell diameters caudally. Despite this pronounced shift, individual motoneuron cell bodies stay aligned with their extending axons. We find that this alignment requires myosin phosphatase activity within motoneurons, and that mutations in the myosin phosphatase subunit *mypt1* increase myosin phosphorylation causing a displacement between motoneuron cell bodies and their axons. Thus, we demonstrate that spinal motoneurons fine-tune their position during axonogenesis and we identify the myosin II regulatory network as a key regulator.

## Author Summary

Embryonic development requires tight coordination between tissues as they frequently grow at different rates. Such differential growth rates can cause shifts between neighboring tissues, and are a particular challenge for individual cells that span multiple tissues, in part because mechanical tension on such cells is predicted to be high. Here we examine how motoneurons whose cell bodies reside in the spinal cord while their axons traverse adjacent tissues compensate for tissue shifts. We find that in zebrafish, motor axons extend into adjacent tissues at a time when both, spinal cord and adjacent tissues grow at different rates and shift positions against each other. Despite this pronounced shift, individual motoneuron cell bodies stay aligned with their extending axons. We demonstrate that the regulatory network of the molecular motor protein myosin II in motor neurons is key for this alignment as mutations in the myosin phosphatase subunit *mypt1* increase myosin phosphorylation and cause a displacement between motoneuron cell bodies and their axons. Movements between spinal cord and adjacent tissues are conserved from fish to humans, and it is therefore likely that similar mechanisms exist in mammals to ensure correct neuronal alignment to compensate for tissue shifts.

## Introduction

It has been long recognized that during embryonic development of multicellular organisms, differential growth rates and morphogenetic movements of adjacent tissues are highly coordinated [1, 2]. For example, the developing vertebral column and the spinal cord exhibit differential growth rates and shift relative to one another [3], suggesting that mechanisms exist to ensure coordinated development between these two anatomically and functionally highly interconnected tissues. The relative shift between the vertebral column and the spinal cord poses a particular challenge for developing motoneurons. While their cell bodies reside in the spinal cord, their axons exit the spinal cord and traverse tissues that grow at a different rate, thus necessitating developmental mechanisms to constantly adjust either axonal projections or cell body positions relative to one another.

Although morphogenetic movements between the developing spinal cord and adjacent tissues are well documented [3], whether axons or cell bodies adjust their position to compensate for tissue shifts has not been examined. Furthermore, the temporal relationship between such tissue shifts relative to when motor neurons extend their axons into adjacent tissues is unknown. The general view is that neuronal migration ceases prior to axon initiation [4, 5], calling into question whether neuronal cell bodies can even adjust their position while their axons are actively growing. Only in a few cases, such as cortical projection neurons [6–8] or brachial facial pioneer motor neurons [9], have neurons been observed to continue their migration after neurite formation. Thus, the cellular and the molecular mechanisms by which developing motoneurons compensate for shifts of tissues through which their axons extend are not well understood.

Here we use live cell imaging to track the alignment of individual spinal motoneurons and their pathfinding axons relative to adjacent notochord cells. We find that after the onset of axonogenesis notochord cells shift their position caudally relative to that of identified motoneurons. Despite a dramatic shift, motoneuron cell bodies remain well aligned with their axons, suggesting an underlying mechanism that enables motoneurons to adjust their position. In fact, we identify myosin phosphatase as a cell intrinsic regulator that ensures compensatory fine tuning of spinal motoneuron position to maintain alignment with their extending motor axons. Combined, our data reveal a previously unappreciated role for the myosin II regulatory network to coordinate synchronized development of adjacent tissues.

## Results

### Spinal motor neurons shift position relative to adjacent notochord cells during axonogenesis

**Fig 1:**
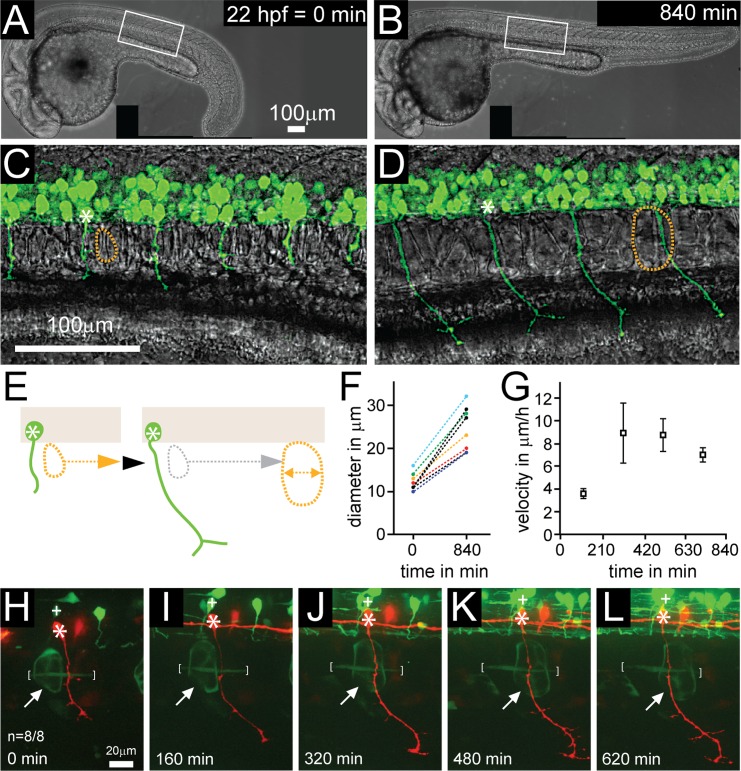
Notochord cell expansion and shift during motor axon outgrowth. (A-D) Time-lapse of a developing zebrafish embryo with transgenic expression of GFP in motoneurons (*mnx1*:*GFP*), starting at 22 hpf for 840 min. (A, B) Low magnification bright field images, generated by stitching together several images. (C, D) Higher magnification images of approximately the boxed areas in A (different embryos), in brightfield and GFP, generated by overlaying substacks containing motor neurons and notochord cells, respectively. While the embryo grows and axons are extending, there is a progressive posterior shift of an ‘identified’ notochord cell (outlined by dashed yellow circle) relative to a GFP positive CaP motoneuron (white asterisk). (E) Schematics of this shift. Note that as CaP motor axons (green) are extending and notochord cells (yellow dotted circle) shift posteriorly, the diameter of notochord cells increases, quantified in (F; n = 9). (G) Velocity of posterior shift of notochord cells relative to motor axons in μm/h over time (n = 6). Shift velocity is initially low and peaks between 210 and 630 min. (H-L) Time-lapse imaging of a CaP motoneuron labeled in red *(mnx1*:*mKate)* in *Evx1*:*Gal4; UAS*:*GFP* double transgenic embryos from 21 hpf until axons have fully extended to the ventral myotome (620 minutes). Note that an adjacent, GFP positive interneuron (+) and adjacent individual muscle fibers (white brackets) both stay aligned with the motoneuron. In contrast, individually labeled notochord cells (white arrow) shift progressively posteriorly compared to the labeled CaP motoneurons (n = 8/8). See also S1 Movie.

Motoneuron cell bodies of the peripheral nervous system reside in the spinal cord while their axons extend into adjacent tissues. Differences in growth rates and hence shifts between the spinal cord relative to adjacent tissues has previously been reported [3], however if and to what extent this process overlaps with the time period when motor axons exit from the spinal cord and migrate through adjacent tissues has not been examined. We used time-lapse imaging to track the position of identified motoneurons relative to that of adjacent tissues during development. In zebrafish, the earliest developing motorneurons, the ventrally projecting caudal primary (CaP) motoneurons begin axonogenesis at around 16–18 hours post fertilization (hpf), and their axons exit the spinal cord at segmental exit points shortly thereafter between 17–19 hpf [10–13]. Over the next 10 hours motor axons pioneer a ventral path, migrating between the adjacent notochord and myotome through an environment rich in extracellular matrix (ECM) produced by both notochord and muscle cells [10, 12–14]. Time-lapse analysis starting at 22 hpf revealed that as GFP labeled CaP motor axons pioneer the ventral path, notochord cells expand and progressively shift caudally relative to the position of individual CaP motoneurons (Fig 1A–1G). This shift occurred initially at a rate of 3.6 ± 0.43 μm/hour and increased over time to a rate of up to 8.9 ± 2.6 μm/hour (Fig 1G). Over a 14 hour time period this resulted in a significant shift (98.6 ± 5.0 μm) between individual CaP neurons and notochord cells. Concomitantly with this posterior shift, the diameter of individual notochord cells increased (Fig 1C–1E, quantified in 1F), reflecting the process of vacuole inflation and expansion within notochord cells [15].

Since this tissue shift has not been previously been documented in zebrafish, we used more definitive cellular markers to further characterize this process. To that end, we labeled individual CaP motoneuron using *mnx1*:*mKate* in *Evx1*:*Gal4; UAS*:*GFP* transgenic embryos [16]. In these embryos, interneurons and occasionally notochord and myotomal muscle cells are labeled with GFP, allowing singly labeled motoneurons to be traced over time in relation to singly labeled notochord and muscle cells. Time-lapse analyses confirmed a significant shift between CaP neurons and GFP positive notochord cells (Fig 1H–1L and S1 Movie). Importantly, the position of cell bodies of individual GFP positive interneurons relative to CaP cell bodies stayed constant, suggesting that the entire spinal cord shifts relative to the adjacent notochord (n = 8/8, Fig 1H–1L and S1 Movie). In contrast to notochord cells, myotomal muscle cells stayed aligned with CaP motoneurons (Fig 1H–1L and S1 Movie). Therefore, we focused on the shift between individual CaP motoneurons and adjacent notochord cells. Despite this dramatic shift between the spinal cord and adjacent tissues, individual CaP cell bodies remained well aligned with their axons, suggesting the existence of compensatory mechanisms to prevent a shift between motoneuron cell bodies and their axons.

### *Mypt1* myosin phosphatase mutants exhibit motoneuron defects

**Fig 2:**
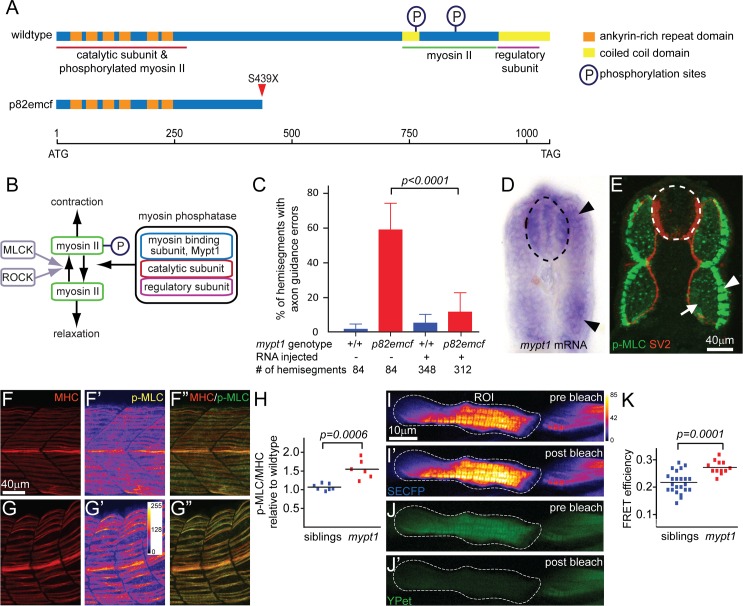
A non-sense mutation in *mypt1* causes axon guidance errors. (A) Schematics outlining the domains of the wildtype (top) and the truncated *mypt1*^*p82emcf*^ protein (bottom). (B) Phosphorylation-dependent regulation of myosin II: Myosin light chain kinase (MLCK) and Rho kinase (ROCK) increase myosin II phosphorylation and thereby enhance myosin II contractility. Conversely, Myosin phosphatase composed of Mypt1, catalytic and regulatory subunits decreases myosin II phosphorylation and thus causes myosin II relaxation. (C) Injection of 250pg wildtype *mypt1* mRNA into one-cell stage wildtype (blue bars) and *p82emcf* mutant embryos (red bars) significantly reduced motor axon guidance errors in *mypt1* mutant embryos as assayed at 26 hpf using SV2 staining (p<0.001; two-tailed t-test). (D) In 25 hpf embryos *mypt1* mRNA is readily detectable in the spinal cord (dotted line) and in the myotomes (arrowheads). (E) 3D projection image of a cross section through the trunk of a 26 hpf embryo reveals p-MLC expression in slow-twitch muscle cells (green, arrow head) and in fast-twitch muscle fibers (arrow), while in the spinal cord (dashed circle) p-MLC expression levels are below detection limit. Motor axons are stained with SV2 (in red). (F-H) Co-staining of MHC and p-MLC in siblings (F) and *mypt1* mutants (G). Compared to MHC levels (F, G), p-MLC levels are increased in *mypt1* mutants (G’, G”) when compared to wildtype (F’, F”; quantified in H). (I-K) FRET analysis using the SECFP donor (I pre, I’ post bleaching) and the YPet acceptor (J pre, J’ post bleaching) reveals increased MLC phosphorylation, quantified in K. Note that non-bleached areas (outside of the region of interest, ROI) remained unchanged.

To define genetic entry points into how motoneurons compensate for tissue shifts we examined a collection of mutants with defects in neuromuscular connectivity [17]. One of these mutants, *p82emcf*, displays severe motor axon branching defects [17], as well as incorrectly positioned motoneuron cell bodies (see below). To identify the causative mutation underlying the *p82emcf* phenotype, we employed a positional cloning strategy and mapped the mutation to a ~350 kb region on chromosome 4, which contains three annotated genes. Genomic and cDNA sequencing revealed a non-sense mutation in one of the three genes encoding the myosin-binding subunit of myosin phosphatase, ***p***rotein ***p***hos***p***hatase ***1***, ***r***egulatory subunit ***12a*** (***ppp1r12a***) commonly known as *mypt1*. *Mypt1* acts as a binding platform by assembling the regulatory and catalytic subunits of the myosin phosphatase complex, and by recruiting substrates such as phosphorylated myosin II, to the complex (Fig 2A) [18, 19]. Myosin phosphatase-dependent dephosphorylation of myosin II reduces actomyosin contractility (Fig 2B) [18, 19]. While myosin II activation via myosin light chain kinase (MLCK) and Rho-associated protein kinase (ROCK) [20] are well known to regulate motoneuron development and function [21, 22], the role of myosin phosphatase in motoneuron development has not been examined in great detail. Zebrafish *mypt1* mutants have previously been identified based on defects in liver organogenesis, astroglial development, axonal pathfinding, and brain morphogenesis [23–25].

The *mypt1* C1316A mutation introduces a premature stop after 438 amino acids, thereby severely truncating the wildtype protein (1049aa) and deleting key phosphorylation residues as well as the binding site for the regulatory subunit (Fig 2A) [18, 19]. The truncated mutant protein is likely to severely reduce or abolish myosin phosphatase activity, predicted to significantly enhance myosin II activity (Fig 2B) [24, 26]. Although we cannot exclude the possibility that the truncated mutant protein can act as a dominant negative version of *mypt1* when overexpressed, heterozygous *p82emcf* embryos do not display obvious motoneuron defects. To confirm that the mutation in *mypt1* causes the *p82emcf* mutant phenotype, we performed mRNA rescue experiments. One-cell stage embryos were injected with wildtype *mypt1* mRNA, and embryos were scored at 26 hpf for motor axon defects. Injection of wildtype *mypt1* mRNA into *p82emcf* mutant embryos reduced motor axon guidance defects from 58% to 11% (Fig 2C), providing compelling evidence that *mypt1* encodes the gene mutated in *p82emcf* animals.

To determine whether mutations in *mypt1* indeed increase or prolong myosin II activity in zebrafish embryos, we examined the phosphorylation status of a myosin phosphatase substrate, phosphorylated myosin light chain (p-MLC) [18]. *Mypt1* is widely expressed in the zebrafish embryo [23], and at 25 hpf *mypt1* mRNA and p-MLC are both readily detectable in slow-twitch skeletal muscle cells (Fig 2D and 2E). Quantification of p-MLC in slow-twitch skeletal muscle cells revealed significantly higher levels in *mypt1* mutants compared to wildtype siblings (Fig 2F–2H). Furthermore, expression of a fluorescence resonance energy transfer (FRET) based biosensor for myosin light chain phosphorylation [27] in individual muscle cells confirmed that in *mypt1* mutants myosin light chain phosphorylation is increased (Fig 2I–2K). Thus, mutations in *mypt1* lead to elevated phosphorylation of myosin light chain, and cause severe motor axon guidance defects.

### *Mypt1* regulates CaP axon branching through a cell-non autonomous mechanism

**Fig 3:**
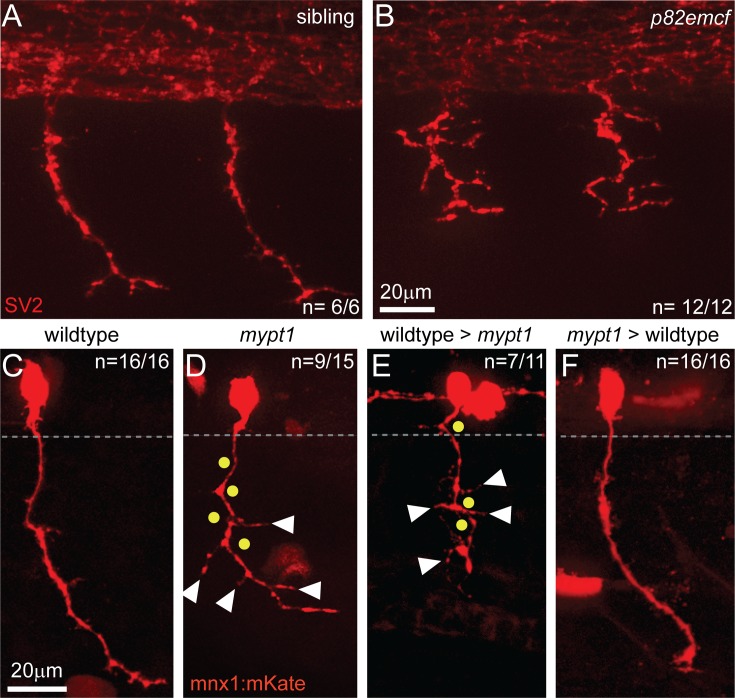
Mypt1 is required in the environment to restrict excessive axon branching. (A, B) SV2 antibody labeling at 25 hpf reveals disorganized motor axon morphologies in *mypt1*^*p82emcf*^ mutants (B) compared to wildtype siblings (A). Unlike wildtype CaP motoneurons (C), individually labeled mutant motor axons (using *mnx1*:*mKate)* project with frequent changes in projection direction (dots), and exhibit excessive branching (arrowheads). The gray dashed line marks the border of the spinal cord. (E, F) Transplantation of rhodamine-dextran labeled wildtype blastula stage cells into *mypt1* mutants analyzed at 26–27 hpf exhibit mutant motor axon phenotypes (E). Conversely, transplantation of mutant motoneurons into a wildtype host results in wildtype-like motor axon projections (F).

We initially identified *mypt1* (*p82emcf*) mutants on the basis of a strong motor axonal phenotype (Fig 3A and 3B) [17]. To understand how *mypt1* and myosin phosphatase activity influence motoneuron development, we first asked whether they regulate axon-axon fasciculation or axonal branching, as defects in either of these processes can result in the axonal phenotypes observed (Fig 3A and 3B). To distinguish between these possibilities, we performed single cell labeling of individual primary motor neurons. We focused our analysis on ventrally projecting CaP motoneurons [12]. In 26 hpf wildtype embryos, CaP motoneurons (n = 16/16) displayed their typical axonal morphology [10, 12, 28, 29]. In contrast, in *mypt1* mutants CaP axons were excessively branched and/or displayed zigzag-like projections (n = 9/15; p = 0.002), consistent with the idea that *mypt1* and myosin phosphatase activity regulate motor axon branching (Fig 3C and 3D). Importantly, in *mypt1* mutants motoneuron specification is unaffected (S1 Fig).

We next asked whether *mypt1* acts within motoneurons or in their environment to restrict excessive axonal branching. Chimera analysis revealed that wildtype-derived motoneurons when transplanted into *mypt1* mutant hosts displayed the excessive branching phenotype characteristic for mutants (n = 7/11, Fig 3E). Conversely, *mypt1*-derived motoneurons transplanted into wildtype hosts developed wildtype-like axons (n = 16/16, p = 0.0004, Fig 3F), demonstrating that *mypt1* functions in the environment of motoneurons to regulate axonal branching. Thus, *mypt1* suppresses excessive axonal branching through a cell non-autonomous mechanism. We have previously shown that in zebrafish a distinct subset of muscle cells, the slow-twitch or adaxial muscle cells delineate the future motor axonal path, and that they provide cues critical for motor axon guidance and branching [30–32]. We therefore examined adaxial cell growth and differentiation in *mypt1* mutants. This revealed that in *mypt1* mutants adaxial muscle fibers display reduced growth, yet are properly polarized and differentiated, and form synaptic contacts with motor axons (S2 Fig). Thus, *mypt1* might regulate muscle fiber growth and axonal branching through a common, muscle intrinsic mechanism.

### *Mypt1* controls motoneuron positioning cell-autonomously

**Fig 4:**
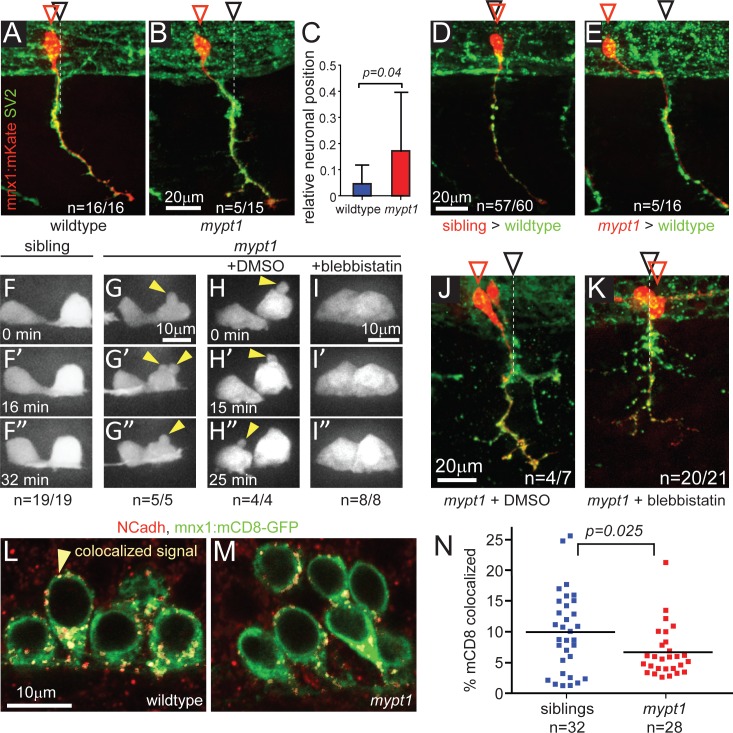
*mypt1* is required cell-autonomously for motoneuronal positioning. (A-C) SV2 staining (all axons, green) combined with stochastically labeled CaP motor neurons using *mnx1*:*mKate* (single cell labeling, red) to determine relative CaP soma positions in 26 hpf wildtype (A) and *mypt1* mutant embryos (B). Red arrowheads indicate the position of CaP cell bodies, black arrowheads the position of the axonal exit point. In contrast to wildtype, 33% of mutant CaP cell bodies were shifted rostrally (p = 0.0177, Fisher exact; for detail on quantification, see Material and Methods). Bar graph of relative neuronal position in wildtype and *mypt1* mutants (C). Following transplantation, wildtype-derived CaP motoneurons (labeled with rhodamine-dextran, red) exhibited normal motoneuron positioning in 26–27 hpf wildtype embryos (D). In contrast, *mypt1* mutant derived CaP motoneuron when transplated into wildtype embryos frequently failed to adjust their position (E; p = 0.0087, Fisher exact). (F-I) Time-lapse analysis of motoneuron membrane dynamics in 18–19 hpf transgenic *mnx1*:*mKate-mnx1*:*mCD8-mKate* embryos, expressing mKate in the cytoplasm and on cell membranes of motoneurons. Motoneurons in *mypt1* mutants displayed membrane blebbing (yellow arrowheads in G, G', G"; n = 5; p = 0.0001, Fisher exact), not observed in wildtype siblings (n = 19, F, F', F"). Treatment with the myosin II inhibitor blebbistatin but not with DMSO (H, H', H") completely abolished membrane blebbing in *mypt1* mutants (I, I', I"; n = 8,). (J, K) SV2 staining (all axons, green) combined with stochastically labeled CaP motor neurons using *mnx1*:*mKate* (single cell labeling, red) to determine relative CaP soma positions in 26 hpf *mypt1* mutant embryos treated with DMSO for control (J) or blebbistatin (K). Blebbistatin treatment significantly reduced rostral mispositioning of *mypt1* mutant CaP motoneurons (p = 0.0077, Fisher exact). (L-N) N-Cadherin (NCadh, red) staining of fixed embryos carrying the *mnx1*:*mCD8-GFP* transgene (green) which labels motoneuronal membranes. Single plane of confocal images with pseudocolored colocalizing pixels generated by Imaris software in a sibling (L) and a *mypt1* mutant (M). Percentage of the green volume (mCD8+) which is colocalized is determined to quantify the fraction of NCadh+ cell membrane (N).

Myosin activity has a well-established role in neuronal cell migration [33–35], prompting us to examine whether in *mypt1* mutants motoneuron migration and/or positioning of motoneurons is affected. Before onset of axon initiation at ~16 hpf, CaP motoneurons migrate in response to semaphorin-neuropilin signaling, moving towards the future segmental spinal cord exit point, where motor axons exit from the spinal cord [5, 36]. At 26 hpf CaP cell bodies have reached their position directly above the segmental exit point (Fig 4A) [10, 28, 29, 30, 37]. In contrast, in *mypt1* mutants, 33% of CaP cell bodies were shifted rostrally relative to the axon exit point (n = 5/15, p = 0.0177), supporting the idea that *mypt1* regulates CaP migration and/or positioning (Fig 4B and 4C).

Given that *mypt1* controls CaP axon branching through a cell non-autonomous mechanism, we wondered whether such mechanism also controls CaP migration/positioning. Chimera analyses revealed that CaP neurons derived from wildtype or heterozygous siblings transplanted into wildtype hosts were mostly correctly positioned (95%, n = 57/60), while cell bodies of *mypt1* mutant motoneurons transplanted into wildtype hosts were frequently shifted rostrally relative to the axon exit point (n = 5/16, p = 0.0087). Thus, chimera analyses demonstrate that unlike axonal branching, CaP migration/positioning requires *mypt1* function intrinsically (Fig 4D and 4E).

Previous studies have shown that in the early zebrafish embryo activation of myosin II or *mypt1* knockdown induces cell-autonomous membrane blebbing [38]. In fact, using a transgenic line expressing a membrane tagged fluorophore in motoneurons to monitor CaP membrane dynamics, we find that at 18–19 hpf *mypt1* mutant compared to wildtype CaP motoneurons exhibited excessive membrane blebbing (Fig 4F and 4G). To demonstrate that this blebbing was indeed caused by enhanced myosin II activity, we treated embryos with the myosin II inhibitor blebbistatin. Blebbistatin treatment abolished membrane blebbing in *mypt1* mutants (Fig 4H and 4I). Furthermore, blebbistatin treatment restored proper positioning of CaP motoneurons in *mypt1* mutants (Fig 4J and 4K), further supporting the notion that enhanced myosin II activity causes neuronal mispositioning. Increased blebbing and mispositioning can be caused by a reduction in cell adhesion, and consistent with this notion, myosin II is central in the control of cell adhesions [39, 40]. Analysis of cell-cell contacts using an N-cadherin antibody revealed a slight but significant decrease in N-cadherin positive cell-cell contacts on *mypt1*-deficient motoneurons (Fig 4L–4N). Thus, loss of *mypt1* function in CaP motoneurons leads to increased myosin II activity which in turn causes excessive membrane blebbing, reduced N-cadherin positive cell-cell contacts and aberrant cell body positioning. However, if and to which extent the reduction of N-cadherin positive cell-cell contacts on motoneuron contributes to the cell migration defects observed in *mypt1* mutants remains unclear.

### *Mypt1* maintains alignment between motoneuron cell bodies and their axons

**Fig 5:**
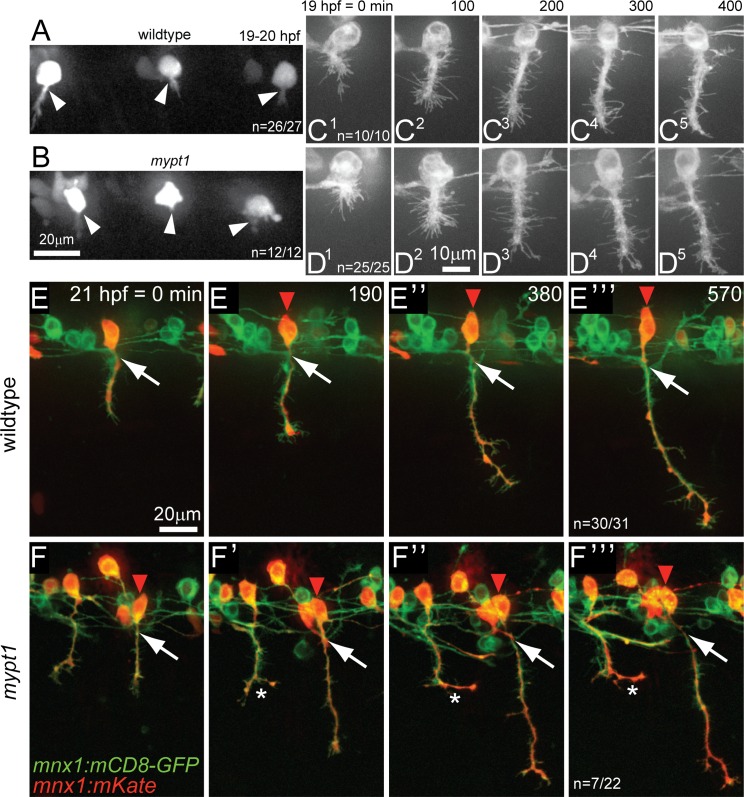
Mypt1 maintains motor neuron position. At the onset of axonogenesis at ~19–20 hp CaP motoneurons in wildtype (A) and *mypt1* mutant (B) embryos carrying the *mnx1*:*mKate mnx1*:*mCD8-mKate* transgene are located at the points where motor axons exit from the spinal cord (n = 26/27 CaP in wildtype, and n = 12/12 CaP in *mypt1* mutants; arrowheads point to the nascent axons). Time-lapse imaging of CaP motoneurons from the onset of axon initiation at 19 hpf until the axons reached the horizontal myoseptum in *mnx1*:*mCD8-GFP* transgenic wildtype (C^1–5^) and *mypt1* mutant embryos (D^1–5^). During the length of the movie (400 min) all wildtype (n = 10/10) and all mutant (n = 25/25) motoneurons retained their position above the spinal cord exit point. Time-lapse imaging of CaP motoneurons labeled with *mnx1*:*mKate* in *mnx1*:*mCD8-GFP* transgenic siblings (E' to E‴) and *mypt1* mutant embryos (F' to F‴) after axons have reached the horizontal myoseptum at 21 hpf until axons have reached the ventral extent of the myotome. While 30/31 wildtype CaP motoneuron cell bodies (red arrowhead) stayed precisely above the exit point (white arrow), 32% (n = 7/22) *mypt1* mutant motoneuron cell bodies shifted progressively rostrally (p = 0.006, Fisher exact). A stalling *mypt1* mutant axon is seen in an adjacent hemisegment (white star in F).

Given that excessive membrane blebbing was detectable already at 16 hpf, we next asked whether *mypt1* is required for CaP’s initial migration towards the segmental axon exit point [5, 36]. For this we performed time-lapse analyses of fluorescently labeled CaP motoneurons from the time of their first appearance in the ventral spinal cord (~16 hpf), through the time period when CaP axons navigate the space outside the spinal cord between the adjacent notochord and somitic muscle cells, up to the point when axons reached the ventral extent of the myotome (~29 hpf; Fig 5). This revealed that at the onset of axonogenesis, *mypt1* CaP somata like those in wildtype were positioned just above the segmental exit point (Fig 5A and 5B), suggesting that in *mypt1* mutants the initial migration of CaP motoneurons towards the segmental exit point is unaffected. Similarly, during the early stages of axon outgrowth, *mypt1* CaP motoneuron cell bodies remained at their correct positions directly above the exit point (Fig 5C and 5D). However, around 24 hpf as CaP axons had extended further towards their synaptic targets, we first noticed that unlike wildtype *mypt1* CaP motoneuron cell bodies shifted rostrally relative to their axons (Fig 5F’). By ~30 hpf, when motor axons have exited form the spinal cord and extended through notochord derived ECM towards the ventral myotome, 30% of *mypt1* mutant CaP motoneuron displayed this rostral shift (n = 7/22, p = 0.006, Fig 5E and 5F). Thus, *mypt1* is dispensable for initial CaP cell body positioning, and instead is required to maintain alignment between the cell body and its axon, once axons have exited from the spinal cord and are well underway towards their synaptic targets.

## Discussion

### Live cell imaging reveals fine-tuning of motoneuron position to compensate for tissue shifts

Similar to other vertebrates, spinal motoneurons in zebrafish develop in register with and thus localize to the same anterior-posterior level as the muscle they innervate [12, 37]. Our live cell imaging revealed a pronounced shift between spinal motoneurons and adjacent mesodermal notochord cells. Such differential or ‘allometric’ growth of the spinal cord relative to mesodermal derivatives has been previously documented in mammalian and human embryos [3, 41, 42]. What had not been previously appreciated is that these tissue movements occur during the time period when motor axons have already exited from the spinal cord and migrate through extracellular space between the adjacent notochord and somite muscle cells (Fig 1). Shifting spinal cord positions relative to adjacent tissues through which motor axons extend is predicted to generate significant mechanical tension between motoneuron soma and their axon, thus necessitating a compensatory mechanisms to keep both aligned and in register.

In zebrafish, the initial migration of spinal motoneurons ceases just before the onset of axon initiation [5, 36]. Here, we identify a second period of motoneuron position fine-tuning at a time when axons have already exited from the spinal cord to innervate their muscle targets. Based on loss of function phenotypes, the early migration requires Semaphorin-Neuropilin but is independent of *mypt1*, while the late fine-tuning appears independent of Semaphorin-Neuropilin [5, 36], but requires *mypt1* function (Fig 5). Thus, *mypt1*-dependent fine-tuning of motoneuron position defines a previously unknown mechanism to compensate for morphogenetic tissue movements between the spinal cord and adjacent tissues that occur well after the onset of axonogenesis.

### A novel role for *mypt1* in fine-tuning neuron cell body position

*Mypt1* has well documented roles in maintaining epithelial integrity [24, 43], modulating smooth muscle contractility [44] as well as regulating cell motility in general [38, 45]. In the nervous system *mypt1* regulates neuronal migration [46], axonal patterning and glial morphology [25]. For many of these processes *mypt1* has been shown to act cell-autonomously [43, 46], while a cell non-autonomous function for *mypt1* has been reported during zebrafish gastrulation [38]. Similarly, we find that *mypt1* acts cell non-autonomously to suppress exuberant axonal branching, possibly through myosin II as we detect increased p-MLC activity in slow-twitch skeletal muscle cells (Fig 2F–2K), which during motor axon outgrowth delineate their migratory path [30].

Conversely, *mypt1* acts cell autonomously within motoneurons as cell bodies migrate posteriorly to stay aligned with their axons as they shift posteriorly with the notochord (Fig 5). Motoneuron membrane blebbing and neuronal mispositioning in *mypt1* are both sensitive to the myosin II inhibitor blebbistatin, demonstrating that *mypt1* regulates fine tuning of motoneuron position through myosin II-dependent actomyosin contractility (Fig 4). Blebbistatin induces severe axonal branching, and thus precluded us to determine whether axonal branching is also regulated via a myosin II-dependent mechanism. Importantly, it is also possible that *mypt1* restricts axonal branching through a myosin II-independent mechanism. Besides regulating myosin II phosphorylation, the MYPT1-containing holoenzyme has also been shown to interact with a large host of putative substrates including moesin, tau, MAP2, Polo like kinase and the transcriptional repressor HDAC7, suggesting that *mypt1* likely has broader functions than myosin regulation [18, 47, 48]. Furthermore, *mypt1* has recently been shown to interact with the insulin receptor substrate-1, and to regulate other pathways including mTOR signaling [49, 50]. Thus, it will be interesting to determine whether *mypt1* restricts axonal branching through a ‘canonical’ pathway such as myosin II or moesin phosphorylation, or through one of these recently identified substrates and pathways.

### Mypt1-dependent motoneuron positioning during notochord driven axis elongation

Our analyses of *mypt1* mutants uncover a second period of motoneuron migration during the time period when axons grow outside the spinal cord towards their muscle targets, and when the embryo elongates along its anterior-posterior axis. In zebrafish the driving force for axis elongation is thought to be at least in part caused by changes in notochord cell morphology [15]. The notochord, which is the defining feature of chordates consists of an outer epithelial-like cell layer and an rod like core of cells each containing a single large vacuole that eventually occupies most of the cell volume [51]. As the vacuoles inflate and expand within the cells, the ECM sheath secreted from the outer cell layer restricts radial expansion of the notochord resulting in the elongation along the anterior-posterior axis [15, 52]. Importantly, vacuole inflation coincides with the time period when we observe the shift between identified spinal motoneurons and notochord cells relative to each other (Fig 1A–1G). Notochord vacuoles have been reported in several vertebrate embryos including in mammalian embryos [53], and recent work in zebrafish has identified vacuole acidification and *rab32* mediated endosomal trafficking to be critical for vacuole expansion and body axis elongation [15].

Notochord cells secrete large amounts of ECM that serves as the substratum for migrating motor axons once they exit from the spinal cord. As notochord cells shift caudal, their ECM might ‘drag’ motor axons along caudally, thereby generating tension between the axons and their cell bodies in the spinal cord. It is conceivable that this tension induces *mypt1* function, which acts in motorneurons to fine-tune their position, thereby compensating for the caudal shift of their axons (Fig 4). How then does *mypt1* fine-tune motoneuron position? We show that inhibiting myosin II activity via blebbistatin restores motoneuron fine-tuning in *mypt1* mutants, providing compelling evidence that *mypt1* modulates actomyosin contractility within motoneurons. Our findings thus implicate the myosin II regulatory network, including myosin light chain kinase (MLCK) and Rho-associated protein kinase (ROCK) [20] as key modulators to adjust motoneuron position during axonogenesis. It will be interesting to determine whether notochord expansion coordinates *mypt1*-dependent motoneuron positioning through mechanical forces or changes in gene expression.

Finally, given that tissue shifts of the spinal cord relative to mesodermal derivatives have previously been documented in mammalian embryos [3, 41, 42], it is tempting to speculate that *mypt1*-dependent compensation for such shifts might be a conserved feature of development. In fact loss of *mypt1* in mice leads to embryonic lethality [54], consistent with a potential role in tissue shift compensation.

## Materials and Methods

### Ethics statement

All experiments were conducted according to an Animal Protocol fully approved by the University of Pennsylvania Institutional Animal Care and Use Committee (IACUC) on January 24, 2014, protocol number 803446. Veterinary care is under the supervision of the University Laboratory Animal Resources (ULAR) of the University of Pennsylvania.

### Zebrafish care and strains

Embryos were generated by natural mating as described [55]. Embryos were raised at 25 to 28°C and developmental stages were determined based on previously described criteria [51]. *p82emcf* mutants (ZFIN ID: ZDB-ALT-050323-2) were previously generated by ENU mutagenesis [17, 55]. *Evx1*:*Gal4; UAS*:*GFP* double transgenic fish were kindly provided by Pierre Drapeau [16].

### Immunohistochemistry

Embryos were fixed in 4% paraformaldehyde with 1% DMSO in 0.1 M phosphate buffer, pH 7.4, then dehydrated in methanol and permeabilized for 30 min. in acetone at -20°C or 1mg/ml collagenase for 7 min (NCadh), and rehydrated with incubation buffer (0.2% BSA, 0.5% Triton X 100 in 0.1 M phosphate buffer, pH 7.4). The following primary antibodies were used: anti-SV2 antibody (1:50, Developmental Studies Hybridoma Bank [DSHB]), anti-phospho-myosin light chain 2 (Ser19, 1:20, Cell Signaling Technology), anti-c-myc clone 9E1 (1:600, Sigma) and anti-F59 (1:10, DSHB) [56, 57], anti-En1 clone 4D9 (1:5, DSHB), anti-NCadh (1:100, Abcam ab12221), anti-znp1 (1:200) [58], and for labeling of AChR, we used Alexa 594-coupled α-bungarotoxin (10μg/ml, Molecular Probes). Embryos were washed at least three times in incubation buffer before adding secondary antibodies conjugated with Alexa Fluor 488, 568 or 594 (1:400, Life Technologies). Antibody incubations were performed for 4 h at room temperature or overnight at 4°C. Embryos were mounted in Vectashield mounting medium (Vector laboratories), head tissue was used for genotyping (see below) and samples were viewed and documented as described below.

### Cell transplantation

To generate chimeric embryos, cell transplantations between wildtype and mutant embryos were performed as previously described [59], with the following modifications. Donor one-cell stage embryos were injected with lysine-fixable rhodamine-dextran (MW 3,000, Life Technologies, 5% in 0.2 M KCl). Embryos were released from their chorions using 0.6 mg/ml pronase and 5–10 labeled cells were transplanted between embryos at the oblong or sphere stage. Embryos were raised in E2 until 26–27 hpf, when host embryos were fixed for staining with SV2 antibody and analysis (see below) and donor embryos were genotyped (see below).

### Blebbistatin treatment

To study the effects of blebbistatin on cellular blebbing, embryos were treated with 0.1% DMSO (control) or 100μM blebbistatin (in 0.1% DMSO) starting at 18 hpf, to study the effects of blebbistatin on neuronal positioning from 21–26 hpf with 30μM blebbistatin (in 0.2% DMSO) and then imaged on a spinning disc confocal as described below. All embryos were genotyped for *mypt1*.

### Chromosomal mapping of *p82emcf* and cloning of *mypt1*

We established two mapping crosses between fish carrying the *p82emcf* allele and polymorphic WIK and AB strains. PCR amplification of microsatellite markers on chromosome 4 revealed linkage to markers z9247 (fw primer: 5'-CTG CTT GAA AGC CTG AGG AC-3', rev primer: 5'-TGC CCA TGT TCA TAG CTC TG-3') and z6977 (fw primer: 5'-TGC TAA TTG GGA CAC TGC AA-3', rev primer: 5'-AGA GTG GCA CAC TGG TAA AAC A-3'). A cDNA clone for *mypt1* (ENSDARG00000010784) was generated by RT-PCR amplification from individual wildtype and mutant embryos using the following primer combinations: Ppp2 fw 5'-GTC AGA CGG CAT TTG ACG TAG C-3' and Ppp2 rev 5'-TAC CGG GAC AGC AGG CTG C-3'; Ppp3 fw 5'-CAC ACG GCC TCG AGA AGA TGA-3' and Ppp3 rev 5'- CTA TTT GGA AAG CTT GCT TAT TAC TCG G-3'; Ppp8 fw 5'-ATG AAG ATG GCG GAC GCC AAG C-3' and Ppp8 rev 5'-TCG TCC TTC TTT CCC TCT CCC TCC-3'; Ppp10 fw 5'-AGC AAC CAG GTG ACC ACC C-3' and Ppp10 rev 5'-GCT ACG ATA GCG CGA CCT G-3'. The *p82emcf* mutation was confirmed by amplifying the lesion site from genomic DNA and sequencing of the PCR product using the primer pair Ppp1r12a exon10 fw: 5'-CTG TGT TCC TCA GGT GAG CAC3' and Ppp1r12a exon10 rev: 5'-CCA CTA AAG TAA AGT GCA AGA GAC CT-3'. For cloning of *mypt1* into pCS2+ the following primer pairs were used: Ppp1r12a EcoRI fw 5'-AAT TGA ATT CAC CAT GAA GAT GGC GGA CGC CAA GC-3' and Ppp1r12a SnaBI rev 5'-CCG CTA CGT ACC AGA CTA GCA-3'; Ppp1r12a SnaBI fw 5'-TGC TAG TCT GGT ACG TAG CGG-3' and Ppp1r12a SnaBI rev 5'-TAT GAT ACG TAC TAT TTG GAA AGC TTG CTT ATT ACT CGG-3'. Both PCR products were initially cloned separately into pCS2+ using EcoRI/SnaBI and SnaBI respectively. After sequencing, both pieces of *mypt1* were fused by SnaBI digest and ligation resulting in full length *mypt1* cDNA in pCS2+. To move *mypt1* into pBSII, it was first amplified using Ppp1r12a EcoRI fw short 5'-AAT TGA ATT CAC CAT GAA GAT GGC GGA-3' and Ppp1r12a XmaI stop rev: 5'-ACC CGG GCT ATT TGG AAA GCT TGC TTA TTA CTC G-3' and then cloned using EcoRI and XmaI.

### Genotyping

Wildtype and mutant alleles of *mypt1* were distinguished using dCAPS PCR. GoTaq polymerase (Promega) and the following PCR conditions were used: initial denaturation at 94°C for 3 min., amplification by 40 cycles of 94°C denaturation for 30 s, annealing at 62°C for 30 s, extension at 72°C for 45 s, final extension at 72°C for 10 min. PCR products were separated in 3% agarose gels in SB buffer using a 1:1 mix of ultrapure agarose (Life Technologies) and methaphore agarose (Lonza). The following primers were used: HinfI-fw: 5'-AGG ACA GGA AGG ATG AGT CTC CTG AAT-3' and intronically binding HinfI-rev: 5'- TGA GGG TGA TGA ATA AGT GGT AGG TGA-3'. PCR products were digested with HinfI restriction enzyme overnight.

### Single cell labeling and transgenic lines

24–26 pg of plasmid DNA containing I-SceI/ meganuclease cleavage sites were injected into one-cell stage embryos to generate transgenic lines as described [60]. The following plasmids were used for injections: pI-SceI mnx1:mKate, pI-SceI mnx1:mKate (2 copies), pI-SceI mnx1:mKate_ mnx1:mCD8-mKate, pI-SceI mnx1:mCD8-GFP (2 copies). To generate *mnx1*:*mCD8-GFP* transgenic fish and for some single cell labeling experiments with mnx1:mKate, we used constructs that contained two copies of the *mnx1* enhancer/ promoter in tandem resulting in supra-additive expression levels and higher rates of transgene-expressing transgenic lines.

### Quantification of motoneuron cell body positioning

Throughout this study, we focused on primary motoneurons and distinguished them from the later born secondary motor neurons based on previously established unambiguous criteria: more oval cell body shape, larger diameter, more dorsal localization of cell bodies in the spinal cord and further extended axonal projections [10, 31]. CaP cell body position was quantified as previously described [28, 29].

### Whole-mount in situ hybridization

Antisense riboprobes for detection of *mypt1* and *isl-1* labeled with digoxygenin-UTP were synthesized by in vitro transcription using T3 polymerase (Promega) and DIG-labeling mix (Roche). As templates, we used XbaI linearized pBS isl-1 [61] and BssHII linearized pBSII containing *mypt1*. Probes were hydrolyzed to an average length of 200 bases by limited alkaline hydrolysis using sodium bicarbonate/ carbonate [62]. Whole mount in situ hybridization was performed as described by [63], with the following modifications: (1) acetic anhydride/ triethanolamine treatment was omitted, (2) torula RNA in the hybridization solution was replaced by 1mg/ml glycogen, and (3) hybridizations were carried out at 60–61°C. Blocking and antibody incubation with anti-DIG antibody (1:5000, Roche) were performed in maleic acid buffer containing 2% blocking reagent (Roche). Detection was performed using BM purple (Roche). Stained embryos were dehydrated, viewed on a compound scope (Zeiss) and documented.

### mRNA injections

Capped *mypt1* mRNA was generated using mMessage mMachine Sp6 transcription kit (Life Technologies) and NotI linearized pCS2+ Mypt1-myc as a template as described by the manufacturer. For mRNA purification, phenol-chloroform extraction was performed. 250 pg capped *mypt1* mRNA in 0.1 M KCl containing 0.05–0.1% phenol red were injected into each one-cell stage embryo from pooled clutches derived from incrosses of *mypt1* heterozygous fish. Embryos were fixed and stained at 26 hpf using anti-SV2-antibody and all embryos were genotyped for *mypt1*.

### Imaging, FRET analysis, image processing and data analysis

Fixed and stained embryos mounted in Vectashield (Vector laboratories) were imaged using either a spinning disk (Olympus) or using a laser scanning confocal microscope (Zeiss, LSM710). Maximum intensity projection images of z-stacks were created using Slidebook (3i) or Image J (NIH) software. Live embryos were mounted in 0.7–0.8% agarose in E3 and anesthetized in 0.022% tricaine and kept at 28°C. For quantification of FRET efficiency, donor emission was determined using ZEN software (Zeiss) in a single muscle fiber (region of interest) in which the acceptor was bleached. FRET efficiency was calculated as the ratio of donor emission before minus after acceptor bleaching divided by the emission after bleaching (Fig 2I–2K). Confocal images were further processed and analyzed using the Image J software package (NIH). Image manipulations included adjustment of brightness, contrast, gamma-value, background substraction. Manipulations were always applied to the entire image and to all images in one experiment ensuring that the content of the image wasn't altered. Images were exported and further processed in Photoshop CS4 and final versions of the figures for the manuscript were prepared using Illustrator CS4 and Photoshop CS4 (Adobe). To visualize the entire growing embryo in brightfield (Fig 1A and 1B), several separate maximum projection images were stitched together using Image J (NIH). For better visualization of the notochord cells in brightfield (Fig 1C and 1D), we generated separate substacks, one containing the motor neurons on one side of the fish (GFP) and the other containing the notochord cells (brightfield), generated maximum intensity projection images, merged the channels using Image J (NIH) and used the unsharp mask filter in Illustrator CS4 (Adobe). For Fig 2E, a 3D project of a 36 μm substack was generated in Image J (NIH) and a slightly turned view is shown. For quantification of anti-phospho-myosin light chain 2 staining compared to F59 staining (Fig 2F–2H), we processed all embryos in the same tube during antibody staining, and always imaged the same hemisegments; substacks of 40 μm were subjected to a z-projection (slice summation) and integrated densities were determined after splitting the two channels. For anti-phospho-myosin light chain 2 (p-MLC) staining, outliers were removed to reduce noise using Image J (NIH). Values in Fig 2H represent the ratio of p-MLC/ F59 relative to the ratio in wildtype sibling embryos. For better visualization of the dynamic range, p-MLC signal and SECFP channel for FRET were changed to a thermal color scale (Fig 2F', 2G', 2I and 2I'). N-Cadherin colocalization with the neuronal membrane marker mCD8-GFP was determined using the colocalization tool within Imaris software (Bitplane). Colocalized pixels were pseudocolored by the software to help visualizing colocalization and colocalization was quantified as the percentage of the green channel volume above threshold which is colocalized with the red channel (Fig 4L–4N). Statistical analysis was performed using Prism 5 (GraphPad) and all data are presented as mean ± standard deviation. P values were calculated using either a 2-tailed student's t-test for continuous and normally distributed data or Fisher exact test for categorical outcomes using Prism 5 or a Graph Pad web tool (GraphPad).

## Supporting Information

S1 Movie Notochord cell shift during motor axon outgrowth. Time-lapse imaging of a CaP motoneuron labeled in red *(mnx1*:*mKate)* in *Evx1*:*Gal4; UAS*:*GFP* double transgenic embryos from 21 hpf until axons have fully extended to the ventral myotome (620 minutes). Adjacent GFP positive interneurons and an adjacent individual muscle fiber stay aligned with the motoneuron while individually labeled notochord cells shift progressively posteriorly compared to the labeled CaP motoneuron.
(MOV)

S1 Fig *Mypt1* is dispensable for motoneuron specification. (A, B) *In situ* hybridization for *isl-1* in 25 hpf embryos. Rohon Beard neurons in the dorsal spinal cord and motoneurons in the ventral spinal cord both express *isl-1* in both, siblings (A) and *p82emcf* mutants (B). Higher magnification images of the orange-boxed area in the ventral spinal cord containing motoneurons on the right side: sibling (A') and *p82emcf* mutant (B'), demonstrating normal neuronal specification.
(TIF)

S2 Fig Mypt1 is dispensable for polarity and postsynaptic differentiation of adaxial /slow twitch muscle cells but is required for muscle cell growth. (A, B) Immunostaining for Engrailed-1 (En1, red) and axonal Znp1 (green) at 26 hpf in wildtype (A) and *mypt1* mutant embryos (B), showing normal localization of En1 positive elongated nuclei of adaxial muscle cells in the anterior somites (anterior of the motor axons, arrowheads). This indicates normal specification and polarity of adaxial muscle cells in *mypt1* mutant embryos. (C, D) Staining with bungarotoxin (BTX, red) and for axonal Znp1 (green) at 26 hpf in wildtype (C) and *mypt1* mutant embryos (D), showing normal sites of postsynaptic differentiation in muscle cells directly opposing motor axons. This indicates normal muscle fiber differentiation in *mypt1* mutant embryos. (E-H) Immunostaining for myosin heavy chain in adaxial muscle cells (F59, red) at 26 hpf in wildtype (E) and *mypt1* mutant embryos (F), showing irregular spacing of muscle cells (stars) and shorter muscle cells in *mypt1* mutant embryos. Quantification of muscle fiber length at 18 hpf and 26 hpf (G) showing that *mypt1* mutant muscle cells have normal length initially, but fail to grow over time. Quantification of sarcomere length at 26 hpf (H) as determined by the interval of myosin heavy chain rich A-bands, showing that the reduced muscle cell length is not caused by sarcomere shortening, but rather by reduced addition of new sarcomeres.
(TIF)

S1 Data Points Data points used to generate graphs. (PDF)
