# Supplementary figures and images for: Myosin phosphatase Fine-tunes Zebrafish Motoneuron Position during Axonogenesis
Source: PLoS Genet. 2016 Nov 17;12(11):e1006440. doi: 10.1371/journal.pgen.1006440 (PMC5147773; doi:10.1371/journal.pgen.1006440)

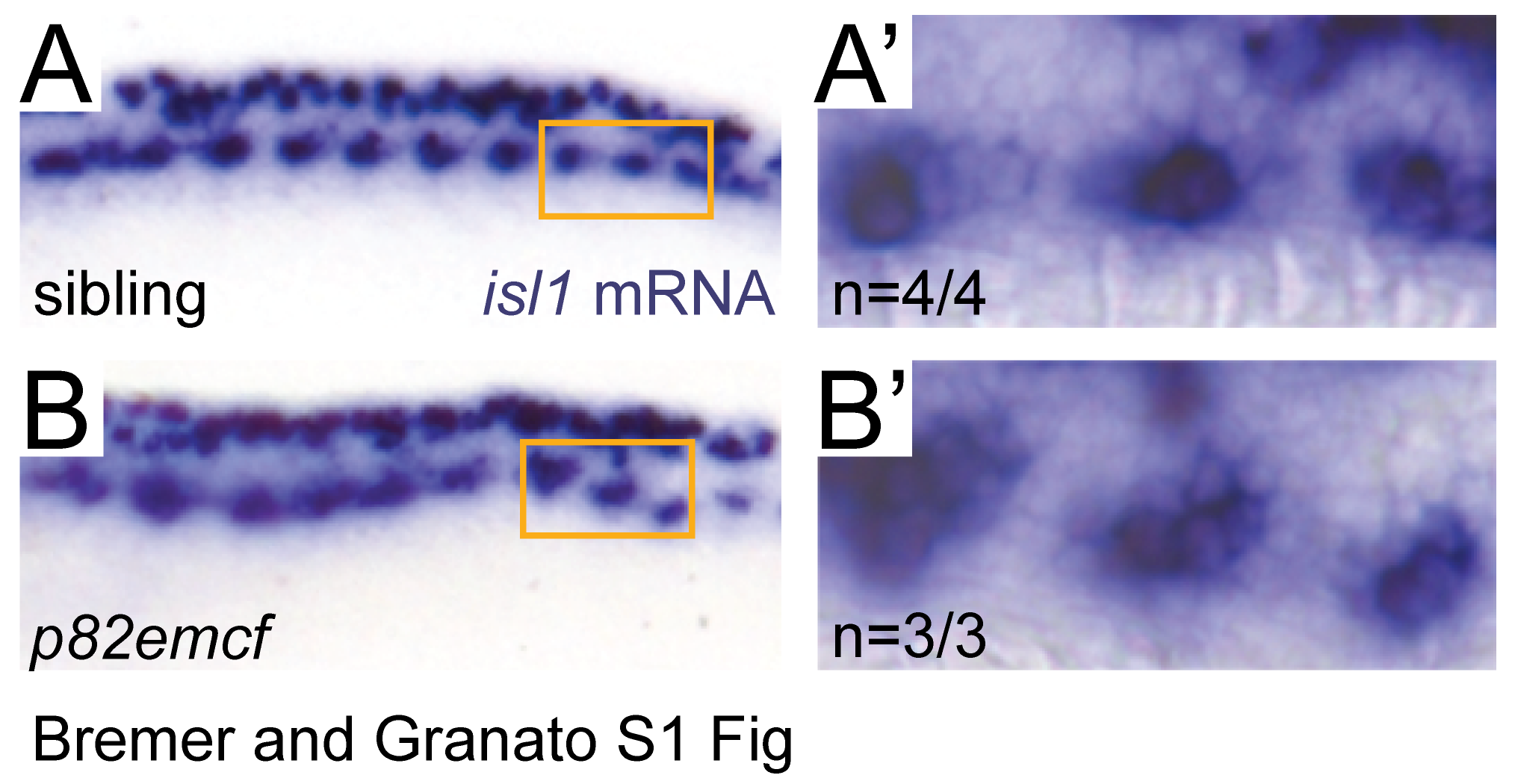

Supplement: S1 Fig — (A, B) In situ hybridization for isl-1 in 25 hpf embryos. Rohon Beard neurons in the dorsal spinal cord and motoneurons in the ventral spinal cord both express isl-1 in both, siblings (A) and p82emcf mutants (B). Higher magnification images of the orange-boxed area in the ventral spinal cord containing motoneurons on the right side: sibling (A') and p82emcf mutant (B'), demonstrating normal neuronal specification. (TIF) [file pgen.1006440.s002.tif]

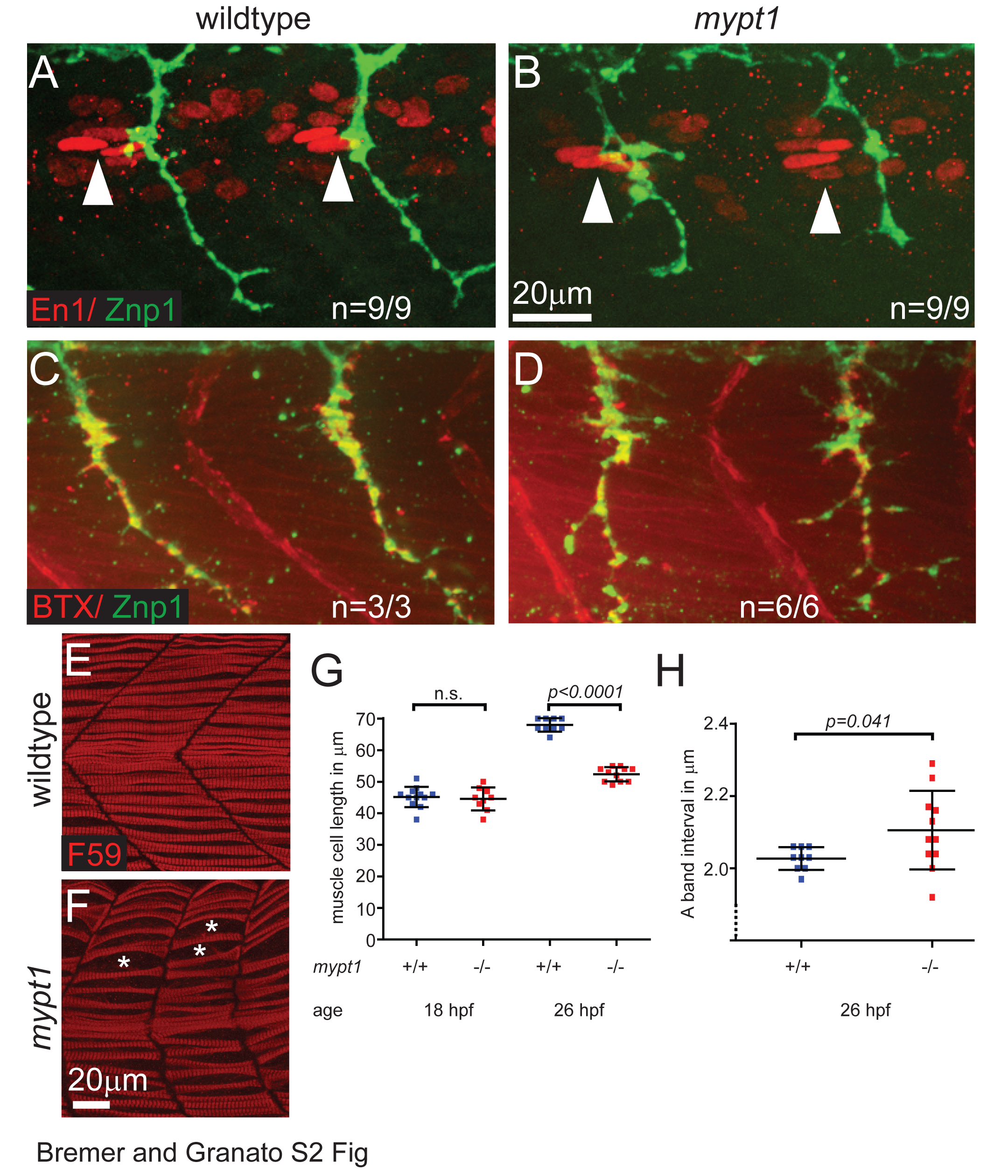

Supplement: S2 Fig — (A, B) Immunostaining for Engrailed-1 (En1, red) and axonal Znp1 (green) at 26 hpf in wildtype (A) and mypt1 mutant embryos (B), showing normal localization of En1 positive elongated nuclei of adaxial muscle cells in the anterior somites (anterior of the motor axons, arrowheads). This indicates normal specification and polarity of adaxial muscle cells in mypt1 mutant embryos. (C, D) Staining with bungarotoxin (BTX, red) and for axonal Znp1 (green) at 26 hpf in wildtype (C) and mypt1 mutant embryos (D), showing normal sites of postsynaptic differentiation in muscle cells directly opposing motor axons. This indicates normal muscle fiber differentiation in mypt1 mutant embryos. (E-H) Immunostaining for myosin heavy chain in adaxial muscle cells (F59, red) at 26 hpf in wildtype (E) and mypt1 mutant embryos (F), showing irregular spacing of muscle cells (stars) and shorter muscle cells in mypt1 mutant embryos. Quantification of muscle fiber length at 18 hpf and 26 hpf (G) showing that mypt1 mutant muscle cells have normal length initially, but fail to grow over time. Quantification of sarcomere length at 26 hpf (H) as determined by the interval of myosin heavy chain rich A-bands, showing that the reduced muscle cell length is not caused by sarcomere shortening, but rather by reduced addition of new sarcomeres. (TIF) [file pgen.1006440.s003.tif]
