# Supplementary material for: Myosin phosphatase Fine-tunes Zebrafish Motoneuron Position during Axonogenesis
Source: PLoS Genet. 2016 Nov 17;12(11):e1006440. doi: 10.1371/journal.pgen.1006440 (PMC5147773; doi:10.1371/journal.pgen.1006440)
Supplement: S1 Data Points — (PDF) [file pgen.1006440.s004.pdf]

Notochord cell expansion
(see Fig 1F)

	diameter in μm at 0 min	diameter in μm at 840 min
**embryo 1 cell 1**	16	32
**embryo 1 cell 2**	11	29
**embryo 1 cell 3**	11	27
**embryo 2 cell 1**	11	19
**embryo 2 cell 2**	10	19
**embryo 2 cell 3**	14	28
**embryo 3 cell 1**	13	23
**embryo 3 cell 2**	13	23
**embryo 3 cell 3**	12	20

Notochord cell shift
(see Fig 1G)

	shift in μm compared to baseline	shift in μm per h
**embryo 1 cell 1**		
0 min	0	
210 min	14.98	4.28
420 min	53.06	10.88
630 min	77.11	6.87
840 min	102.18	7.16
**embryo 1 cell 2**		
0 min	0	
210 min	12.3	3.51
420 min	46.66	9.82
630 min	77.13	8.71
840 min	98.84	6.20
**embryo 2 cell 1**		
0 min	0	
210 min	10.62	3.03
420 min	49.15	11.01
630 min	77.05	7.97
840 min	98.99	6.27
**embryo 2 cell 2**		
0 min	0	
210 min	13.17	3.76
420 min	50.31	10.61
630 min	78.41	8.03
840 min	105.75	7.81
**embryo 3 cell 1**		
0 min	0	
210 min	12.5	3.57
420 min	30.32	5.09
630 min	67.93	10.75
840 min	93.41	7.28
**embryo 3 cell 2**		
0 min	0	
210 min	11.36	3.25
420 min	32.44	6.02
630 min	67.62	10.05
840 min	92.63	7.15
total shift in μm (mean)	98.6	
standard deviation	5.0	

Percentage of hemisegments with axon guidance defects (see Fig 2C)
with 12 hemisegments evaluated per embryo

**genotype**	**wild type**	***mypt1***	**wild type**	***mypt1***
***mypt1* RNA injected**	**no**	**no**	**yes**	**yes**
embryo 1	0	75	8	25
embryo 2	0	50	0	0
embryo 3	0	67	0	8
embryo 4	8	50	0	16
embryo 5	0	58	0	0
embryo 6	0	75	0	25
embryo 7	0	33	16	25
embryo 8			0	8
embryo 9			8	8
embryo 10			8	0
embryo 11			8	16
embryo 12			8	0
embryo 13			0	0
embryo 14			8	0
embryo 15			8	0
embryo 16			0	0
embryo 17			0	0
embryo 18			8	0
embryo 19			0	8
embryo 20			0	8
embryo 21			0	16
embryo 22			0	16
embryo 23			0	16
embryo 24			8	25
embryo 25			8	33
embryo 26			8	33
embryo 27			8	
embryo 28			8	
embryo 29			16	
mean (%)	1.1	58.3	4.7	11.0
standard deviation	3.0	15.3	5.0	11.2

pMLC/MHC ratio (see Fig 2H)

ratio of fluorescent intensity pMLC to MHC relative to mean value in wild type

	**siblings**	***mypt1***
embryo 1	1.01	1.74
embryo 2	0.96	1.49
embryo 3	1.03	1.55
embryo 4	1.02	1.92
embryo 5	1.19	1.23
embryo 6	1.16	1.35
embryo 7	1.10	
mean	1.07	1.55
standard deviation	0.085	0.25

FRET efficiency (MLC phosphorylation, see Fig 2K)
(fluorescent intensity in ROI post bleaching minus fluorescent intensity in ROI pre bleaching) divided by fluorescent intensity in ROI post bleaching

**siblings**		***mypt1***	
embryo 1 muscle fiber 1	0.23	embryo 1 muscle fiber 1	0.26
embryo 1 muscle fiber 2	0.21	embryo 2 muscle fiber 1	0.26
embryo 1 muscle fiber 3	0.26	embryo 2 muscle fiber 2	0.27
embryo 1 muscle fiber 4	0.18	embryo 3 muscle fiber 1	0.23
embryo 1 muscle fiber 5	0.21	embryo 3 muscle fiber 2	0.25
embryo 1 muscle fiber 6	0.23	embryo 3 muscle fiber 3	0.26
embryo 2 muscle fiber 1	0.23	embryo 4 muscle fiber 1	0.29
embryo 2 muscle fiber 2	0.29	embryo 4 muscle fiber 2	0.26
embryo 2 muscle fiber 3	0.19	embryo 4 muscle fiber 3	0.30
embryo 3 muscle fiber 1	0.20	embryo 5 muscle fiber 1	0.29
embryo 3 muscle fiber 2	0.19	embryo 5 muscle fiber 2	0.28
embryo 3 muscle fiber 3	0.27	embryo 5 muscle fiber 3	0.32
embryo 3 muscle fiber 4	0.24		
embryo 3 muscle fiber 5	0.19		
embryo 3 muscle fiber 6	0.19		
embryo 3 muscle fiber 7	0.14		
embryo 3 muscle fiber 8	0.17		
embryo 3 muscle fiber 9	0.23		
embryo 3 muscle fiber 10	0.28		
embryo 4 muscle fiber 1	0.20		
embryo 4 muscle fiber 2	0.27		
embryo 5 muscle fiber 1	0.16		
embryo 5 muscle fiber 2	0.23		
mean	0.22		0.27
standard deviation	0.040		0.025

Relative position of CaP motoneurons (see Fig 4C)
distance neuronal cell body to axon exit point/ distance between two adjacent axon exit points

	**wildtype**	***mypt1***
	0.12	0.23
	0.11	0.28
	0.063	0.16
	-0.025	0.76
	0.13	0.55
	0.021	0.21
	0.14	0.16
	0.055	-0.049
	-0.037	0.012
	0.061	0.14
	-0.027	-0.048
	0.036	0.0057
	0	0.0034
	0.12	0.079
	-0.11	0.077
	0.069	
mean	0.05	0.17
standard deviation	0.07	0.22

N-Cadherin positive motoneuronal cell surface (Fig 4N)

% of green channel volume above threshold colocalized with red channel

	siblings	*mypt1*
	2.55	6.18
	3.20	3.45
	15.85	2.81
	14.26	4.59
	25.63	3.36
	24.80	3.96
	6.00	5.17
	5.36	6.12
	1.48	10.94
	1.67	10.02
	13.09	5.98
	15.09	8.35
	14.99	4.03
	15.99	4.78
	8.67	5.90
	7.01	2.63
	7.84	10.09
	8.62	4.44
	7.83	13.50
	12.91	21.29
	10.76	5.58
	12.00	12.17
	11.17	3.16
	10.09	4.04
	1.28	3.24
	1.26	6.90
	8.77	6.00
	10.91	7.85
	2.12	
	2.35	
	17.73	
	17.02	
mean	9.95	6.66
standard deviation	6.51	4.09

Muscle cell length in μm
(see S2 Fig)

	siblings (18 hpf)	*mypt1* (18 hpf)	siblings (26 hpf)	*mypt1* (26 hpf)
	45	44	70	54
	51	48	67	50
	45	50	67	50
	48	47	70	52
	47	45	70	55
	46	45	70	49
	43	41	67	53
	42	38	64	54
	45	43	67	50
	46			54
	46			55
	38			
mean	45	45	68	52
standard deviation	3	4	2	2

A band interval in μm at 26 hpf (see S2 Fig)

	**siblings**	***mypt1***
	2.06	2.16
	2.03	2.17
	2.03	1.92
	2.06	2.00
	2.06	2.04
	2.00	2.13
	1.97	2.04
	2.00	2.25
	2.03	2.08
		2.08
		2.29
mean	2.03	2.11
standard deviation	0.03	0.11
